# Pretreatment of hepatetctomized rats with *Coleus forskohlii* did not interfere with the course of hepatic hyperplasia^1^

**DOI:** 10.1590/s0102-865020190060000007

**Published:** 2019-08-19

**Authors:** Pedro Paulo Barros, Gisele Mara Silva Gonçalves, Gustavo Henrique da Silva, Ana Laura Masquetti Fava

**Affiliations:** I PhD, Full Professor, Researcher, Faculty of Pharmaceutical Sciences, Pontifícia Universidade Católica de Campinas (PUC-Campinas), Brazil. Conception of the study, interpretation of data, manuscript writing, critical revision.; II Pharmacist, Faculty of Pharmaceutical Sciences, Fundo de Apoio à Iniciação Científica (FAPIC), PUC-Campinas, Brazil. Design of the study.

**Keywords:** Aspartate Aminotransferases, Alanine Transaminase, Alkaline Phosphatase, Mitosis, Apoptosis, Liver, Rats

## Abstract

**Purpose:**

*Coleus forskohlii* Briq., a medicinal plant originally from India, has been indicated against heart disease, expiratory disorders, convulsions, and hepatic changes, among others. In view of the broad pharmacological potential of the plant and the scarce information about its effects, the objective of the present study was to investigate the effect of its use for pretreatment of partially hepatectomized rats.

**Methods:**

The animals were divided into two experimental groups: Control (CG) receiving physiological saline for 10 days before partial hepatetctomy, and Treated (TG) receiving 40 mg *Coleus forskohlii*/kg/day for 10 days before partial hepatectomy. The treatments were performed by gastric gavage. After the surgical procedure, treatment was continued according to the following groups: CG 24 h, CG 48 h, TG 24 h, and TG 48 hs, and liver tissue and intracardiac blood samples were obtained for histological and biochemical analysis, respectively.

**Results:**

No significant differences were observed in mitotic or apoptotic index or in the concentrations of the enzymes AST, ALT and alkaline phosphatase, and no areas of fibrosis were detected.

**Conclusion:**

Treatment with *Coleus forskohlii* did not interfere with the course of hepatic hyperplasia.

## Introduction

*Coleus forskohlii* Briq., also known as *Plectranthus barbatus*, is a plant species belonging to the family Lamiaceae originating from India, which has been used in traditional medicine against heart disease, respiratory and intestinal disorders, convulsions, and liver alterations^1^. The species is widely diffuse in Brazil, where it is popularly called “Falso-Boldo” or “Brazilian boldo” and is used for the treatment of hepatic and digestive disorders^2^.

Magdah Ganash and Sultan Qanash^3^ conducted studies on *Coleus forskohlii* and *Plectranthus barbatus* in order to quantitate their phenolic acid content and to determine their antitumoral, antioxidant and antimicrobial activity. The authors observed a subtle difference in the activities of the two species possibly due to their different molecular composition and found out that *C. forskohlii* has antioxidant and antibacterial activities against *S. aureus, E.coli* and *S. tiphy.*

Many molecules present in phytotherapeutic agents may have different activities. Ma *et al.* ^4^ determined the anti-inflammatory activity of a *Coleus forskohlii* extract used for the treatment of asthma and of the upper airways due to the modulation of inflammatory cytokines and concluded that this phytotherapeutic compounds can be potentially applied to the treatment of asthmatic conditions. Other studies indicated that the antioxidant potential and other medicinal properties of *Coleus forskohlii* extrat may act as renal protectors and preserving agents against the oxidative deterioration induced by degenerative dieases^5^.

In addition, the extract of *C. forskohlii* roots is known to contain a diterpene with the property of increasing AMPc concentration by activating adenylate cyclase, which results in various therapeutic effects against idiopathic asthma and congestive cardiomyopathy^6^.

Alasbahi and Melzinga^7^described the pharmacological action of forskolin on the cardiovascular system (increasing coronary blood flow), on the reduction of platelet aggregation, on the digestive system (increasing gastric acid secretion and intestinal smooth muscle relaxation), on the respiratory system (bronchodilator effect), on the genitourinary system (relaxation of the detrusor muscle of the bladder and stimulation of renin secretion by the kidneys), on the eyes (reducing intraocular pressure), and on the skin (increasing melatonin production), in addition to antitumoral activity and maintenance of body weight (AMPc-mediated lipolytic effect).

Recent studies have shown that the potential of forskolin to increase AMPc had involved antiproliferative and antimigratory effects against human pancreatic cells^8^.

Guo *et al.* ^9^ demonstrated that forskolin present in *Coleus forskohlii* may have a protective activity against signs and symptoms of ototoxicity induced by the use of cisplatin. According to these authors, forskolin was able to inhibit the activation of the cellular apoptotic mechanism and the production of reactive oxygen species (ROS).

In view of the broad pharmacological potential of *Coleus forskohlii*, the objective of the present study was to investigate the effects of pretreatment of partially hepatectomized rats with this phototherapeutic agent on hepatic hyperplasia, considering that such effects are poorly known.

## Methods

The experimental protocol of the present study was approved by the Ethics Committee for the Use of Animals of Pontifícia Universidade Católica de Campinas (Protocol nº018/2015) and the experiments were conducted according to the Brazilian Directives for the Care and Use of Animals for Teaching or Research Activities^10^.

The methodology of the present study followed that of previous studies by Barros *et.al.* ^11^. Twenty male Wistar rats weighing 250-260 g were obtained from the Multidisciplinary Animal Experimentation Unit (Animal House) of the University of Campinas, and kept in a room with controlled temperature (22 ± 1°C) under a 12 hour light/dark cycle, with free access to pelleted chow (Nuvilab) and filtered water*.*

### Material

A pharmaceutical grade dry *Coleus forskohlii* extract was purchased from a local compounding pharmacy (Ao Pharmaceutico^TM^, Campinas-SP). Vincristine sulfate was obtained from the Libbs Farmaceutica^TM^ Laboratory, and the enzymatic assay kits were obtained from LaborLab^TM^. All other reagents were analytical grade.

### Partial hepatectomy

The animals were divided into groups (n=5) as shown in Table 1. The treatments were performed by daily gastric gavage over a period of 10 days. Next, the animals were submitted to the surgical procedures and treated for 1 or 2 more days according to the group to which they belonged.

**Table 1:** Experimental groups and treatments.

Groups		Treatments
Control	CG 24 h	Physiological saline/10 days before c
	CG 48 h	
Treated	TG 24 h	40 mg *Coleus forskohlii*/kg/day/10 days before partial hepatectomy
	TG 48 h	

CG 24 h and TG 24 h: treatment continued for 24 hours after partial hepatectomy.

CG 48 h and TG 48 h: treatment continued for 48 hours after partial hepatectomy.

For partial hepatectomy, the animals were anesthetized intraperitoneally with xylazine hydrochloride and ketamine, and approximately 70% of hepatic tissue was removed by the method of Higgins and Anderson^12^. The animals then continued to be treated for 24 or 48 hours after surgery and then submitted to removal of hepatic tissue and blood collection. To this end, vincristine sulfate (1 mg/kg) was administered by intraperitoneal injection in order to block the cell cycle of all hepatocytes in the M phase. The remaining hepatic tissue was then removed under anesthesia, intracardiac blood samples were obtained, and the animals were euthanized^11^.

### Histological processing

The procedures were carried out by the method of Barros *et.al.* ^11^. Hepatic tissue fragments were cut into 7 µm-thick slices and stained with hematoxylin-eosin (HE) and by the Feulgen method (FM). Images were captured digitally using a photomicroscope (Nikon Eclipse E200) coupled to a camera (Nikon Colpix 4500) and mitotic and apoptotic figures were quantiated by microscopic analysis.

### Determination of enzymatic activity

The serum enzymatic activities of alanine-aminotransferase (ALT), aspartate-aminotransferase (AST) and alkaline phosphatase (AP) were determined using commercial kits for human samples (LaborLab^TM^) and a UV-VIS spectrophotometer UV-Vis (Varian^TM^)^11^.

### Data analysis

Data are reported based on the number of cells identified by microscopic analysis. Hepatic tissue slides were analyzed in 20 fields which served as the base for the counts of mitotic and apoptotic figures. Morphometry data and AST, ALT and AP were analyzed statistically using the Graph Pad PRISM^TM^ software. The Mann-Whitney test (p<0.05) was used for group comparisons^11^.

## Results

**Figure 1:**
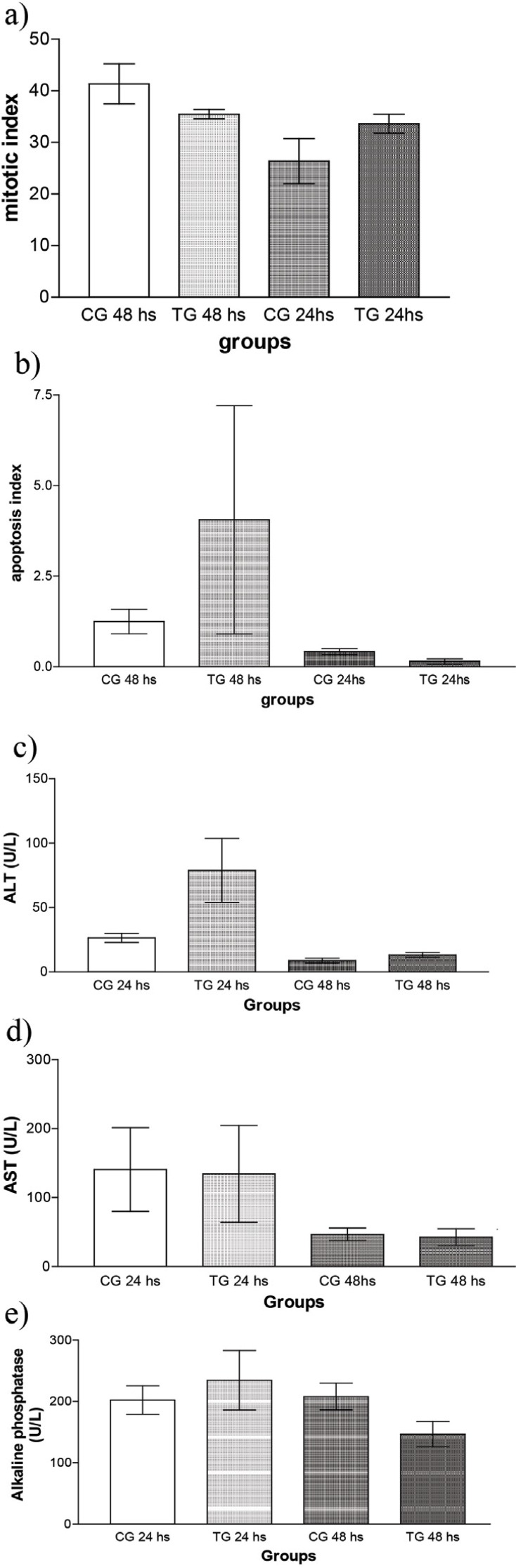
Mitosis (a), apoptosis (b), and enzymatic activity (c-e) parameters of rats submitted to Partial Hepatectomy (CG), and Coleus forskohlii + Partial Hepatectomy (TG). CG 24 hs, CG 48 hs: Groups examined 24 and 48 h after partial hepatectomy, respectively; TG 24 h, TG 48 h: Coleus forskohlii - treated groups examined 24 and 48 h after partial hepatectomy, respectively (n=5).

Data are reported as means and standard deviation. The apoptotic and mitotic indices did not differ significantly from control for either the 24 or 48 our time period after partial hepatectomy (Fig. 1 a,b). Similarly, no difference was observed for the enzymes AST, ALT and AP (Fig. 1 c-e, respectively).

## Discussion

In order to assess the hepatic hyperplasia occurring after treatment with *Coleus forskohlii*, histological analysis was carried out for the calculation of mitotic and apoptotic indices, in view of the fact that partial hepatectomy induces increased mitosis and at the same time represents tissue injury that may induce apoptosis. When the results for the control and treated groups were compared, no significant difference was observed between them, thus showing that treatment with *Coleus forskohlii* did not affect the mitotic or apoptotic index.

On the other hand, Geng *et al.* ^13^ and Jyothi *et al.* ^14^, in a study of the effect of a *Coleus forskohlii* extract on the hepatotoxicity induced by carbon tetrachloride in mice, concluded that this treatment was hepatoprotective. The findings of these authors suggest that the mechanism of action involved in hepatoprotection interfered with the toxicity of carbon tetrachloride. When this result is compared to ours, it can be seen that the hepatoprotection observed by the cited authors was not related to the surgical procedure performed in our study, so that it did not interfere with hepatic hyperplasia after partial hepatectomy.

The data obtained here with the biochemical analyses (AST, ALT and AP) did not show significant differences between the treated and control groups, so that the treatment with *Coleus forskohlii* also did not interfere with the plasma concentrations of these enzymes. This result differed from that reported by Virgona *et al.* ^15^, who supplemented the diet of rats with different concentrations of the *Coleus forskohlii* extract standardized with 10% deforskolin and observed hepatic tissue changes and a dose-dependent increase of plasma AST and ALT. Important results were also obtained by Malarvizhi and Srinivasan^16^, who treated mice with a *C. forskohlii* extract administered orally and confirmed its antitumoral and hepatoprotective activity. In a review study about the toxicity of ingredients of natural origin, Jakopin^17^ reported that the data available for forskolin, the major compound of *Coleus forskohlii,* indicate that this substance appears to be well tolerated and not to be mutagenic. The author did not mention hepatotoxicity. In the present study, despite the absence of a significant difference, the mean values of the apoptotic index and ALT for the group treated at 24 hours (TG 24 h) were higher than their respective controls (CG 24 h). Although consulted authors consider the extract of *C. forskohlii* to be of safe use, these increases may indicate a possible tendency to the occurrence of liver damage, a fact that requires investigation in future studies.

No other studies investigating the relationship between oral administration of a *C. forskohlii* extract and partial hepatectomy were detected in the literature, showing that the present study is original. The treatment with 40 mg/k/day for 10 days used here appears not to have interfered significantly with the hepatic function of treated animals.

It should be pointed out that this plant extract is being commercialized with the objective of aiding weight loss^18^ and that there are data proving that its use together with a low-calorie diet can be useful for the management of metabolic risk factors^19^. However, the extract has shown to induce hepatic drug metabolizing enzymes and to interact with co-administered drugs such as warfarin^20,21^, so that its use should be indicated with caution and under medical supervision.

Although the present study was conducted on experimental animals, while it would be of fundamental importance to conduct clinical studies on humans, the present results suggest that, if a patient who regularly uses this phytotherapeutic agent for other purposes, such as weight loss, for example, should be submitted to a surgical interventionon in the liver, *Coleus forskohlii* probably would not interfere with the functional reestablishment of the liver after the surgery.

## Conclusions

The results obtained after treatment with *Coleus forskohlii* showed that the mitotic and apoptotic indices and the concentrations of AST, ALT and alkaline posphatase did not differ significantly between the experimental animals and their controls. In addition, no areas of fibrosis were observed in the hepatic tissue of treated animals. Thus, we conclude that treatment with *Coleus forskohlii* did not interfere with the course of hepatic hyperplasia. The present study was conducted on experimental animals, but it is of fundamental importance to conduct studies on humans. Our results suggest that *Coleus forskohlii* probably would not interfere with the functional reestablishment of the liver after a surgical intervention.

## Footnotes

1 Research performed at the Laboratory of Biological Tests, Faculty of Nutrition, Pontifícia Universidade Católica de Campinas (PUC-Campinas), Brazil.
